# Letter II

**Published:** 1804-09-01

**Authors:** 


### LETTER II.

### Of Quacks and Empiricism.

### Characters II. and III.

### Dr. Griffenberg. Dr. Mayersbach.

So wonderfully constructed is the human frame, so complicated are the diseases to which it is liable, as to demand the most intense study to acquire the knowledge of one, and long and deep experience to prevent the other. The most sagacious professor of medicine is sensible how inadequate are often his utmost exertions to discover, and consequently to remove, the causes of disease and death. He laudably avails himself of every phænomenon, of symptom, of habit, of weather, of age, of secretions and excretions, and by a scientific combination, is often happy enough to ascertain not only cause and effect, but the remedies which they indicate. Among other means, he will avail himself of the state of the urine, by which much may be suggested in febrile, hepatic, and many other diseases; but, upon this alone, his previous knowledge would not suffer him to depend, if he possess any regard for the welfare of his patient. If, therefore, the best informed physicians find this excretion inadequate to successful practice, it must prove so in a greater and more serious degree with the ignorant impostor; but to such indeed, who confine their practice to the exhibition of one or two nostrums for every disease of the human constitution, it is the same thing, whether they inspect the urine or the spittle, for the inspection is a mere pretence, in order to deceive the ignorant and impose upon credulity. The practice, however, has been sanctioned by antiquity, and and even by persons, whose general information should have shielded them from the arts of imposition. If I mistake not, even the great Burleigh was a dupe to this species of imposition; but this is less surprising than that, at the present day, Lord Chief Baron Macdonald should sanction by his name the Quack Bill for curing worms! This will be the subject of a future letter.

Although the country people have long been deceived by water casters, as they are denominated, their artifices have not for many years produced any general impression upon the citizens of the metropolis till recently, that is, about the year 1772, when Griffenberg, a German Physician, made his appearance in this city; but, although he was a scholar, if not an able physician, he made little progress in delusion, and consequently his poverty made him an easier prey to the vicious propensities of the depraved Lord Baltimore, who bribed him and his wife to act as his pimps, in the attempt to seduce Miss Woodcock. The facts which the trial disclosed ruined the Griffenberg's, although the depraved nobleman escaped the punishment he merited. Miss Woodcock was a virtuous young woman, and lived many years very happily in Bishopsgate-street with Mr. Davis, to whom she was married, and where I have occasionally visited them.

The unfortunate Dr. Griffenberg was a scholar. Whenever I met him, he always addressed me in the Latin language, which was more familiar to him than the English. The very man who hastened his ruin neglected him, and he lived in great distress, and died pennyless, which induced me, from compassion, to be at the expence of his funeral.

From his ashes rose a phœnix of great celebrity, Dr. Mayersbach, near Schweinfurth, in Germany. He came to London in November, 1773; and, from his subsequent success, he must have possessed strong radical powers. Every other scheme that was suggested to his inventive mind having failed, he offered himself to Angelo, who then kept a riding school, but was not accepted, as his diminutive size rendered him unsuitable for an equestrian posture-master. About this period (1773,) he became acquainted, by an introductory letter from Mr. Bresener, his brother-in-law, with his countryman, Dr. Griffenberg, before his reputation was totally blasted by his voluptuous services to Lord Baltimore; and it was agreed between them, that Griffenberg should initiate Mayersbach into his urinary deceptions, for which a share of the profits should be given to the tutor, and which the great success of the pupil was enabled amply to confer; but which was probably withdrawn when Mayersbach became himself a professed adept; at least, so I was informed by Griffenberg and his wife: part of the engagement, indeed, extended to the latter, provided she should survive her husband, which really happened. The agreement, so far as it respected the widow, is literally translated from the original:

"Whereas, Dr. J. T. Griffenberg has, with extraordinary "kindness, shewn me the secrets of his profession, and "thereby put me in a situation to earn my bread as a "doctor, and to succeed in his practice, if I should sur-"vive him; I shall ever consider myself bound by duty "and gratitude to respect the said Dr. Griffenberg as my "parent, and always most punctually to fulfil his will. I "swear before Almighty God, by my soul and salvation, "that if, in the providence of the Most High, I should "survive the said Dr. Griffenberg, that I will always re-"spect his widow; and, as a testimony of my gratitude, "give unto her, during her life, six shillings a week out "of my earnings; in confirmation of which, I hereunto "subscribe my name,

"THEODOR VAN MAYERS

"*London, Nov.* 1773. "OF MAYERSBACH."

At the time that Dr. Mayersbach first came under the tuition of Dr. Griffenberg, he did not know one article of medicine, nor the treatment of one disease, when he published the following quack bill:

"Doctor Van Mayersbach is arrived from Prague, and "intends to remain here some time; he begs leave to "recommend himself to the respectable public, to be "honoured with their confidence, by which he will prove "that he understands the use of medicine, and cures all "inward and outward diseases.

"He tells every person, by his uncommon knowledge "of urine, not only their diseases, but likewise how to "cure them."

The two first patients he had were, one with the itch, and the other with a cough; and he was obliged to place them in another room, till he could receive a message from his master how to proceed. It would have hence been a remuneration which gratitude demanded, independently of written documents, to have relieved the widow; which, however, he absolutely refused, at a time when it was said that his income was at least five thousand pounds a year.

Let

Let it, however, be recorded to Dr. Mayersbach's honour, that in 1773, when he lived in Rupert-street, Goodman's Fields, his wife, after a tedious illness, which proved fatal, had been attended by Johan Toennius, apothecary in Mansell-street; and, on application to Mayersbach in 1776, he faithfully discharged the expence of attendance which her illness had occasioned.

As Mayersbach was totally ignorant of medicines, certain pills, powders, and drops, with directions to give them, under certain circumstances, were sent to him; and these he administered discretionally. As he got a little more fledged, he attempted a loftier flight, and even ventured to handle edged tools; but, in consequence of their indiscriminate use, many serious effects succeeded, which were formally communicated to a board of the Royal College of Physicians, when it was archly observed by one of the board, that the charges merited investigation in the criminal courts of law; and thus the business ended with a laugh at the gentleman who presented these charges, for his ignorance in imagining that the College of Physicians ever did a wise act; or, in any instance, ever promoted medical science.

Mayersbach's reputation continued for some months in the most elevated degree. As a water doctor in the metropolis must be supposed to know more than the water doctors in the country, the devotees to deception flocked to town, or sent up their vials by the stages, and the urinary traffic of the country was transferred to London; and thus the German impostor, who, a few months before could not cure the itch, monopolized the most lucrative professional business in Europe. Among his patients he could claim a Harrington, a Hawke, and even a Garrick.

There was, however, a physician in London hardy enough to attack this popular empiric; and, by his spirited exertions, the delusion was so effectually removed, as to induce the impostor to quit London, and revisit his native continent. In less than twelve months he returned, and was again as much followed as previously to his emigration. The physician who had taken so active a part against the empiric, was dissatisfied with the conduct of the College; he was likewise insulted by a numerous herd of anonymous writers in the public prints; and having become an object of their envy, he avoided further interference; and, with the death of Mayersbach, this species of deception has, in a great measure, lost its influence in London, and new modes of deception have been practised

 by by a new race of German empirics; at the head of which may be placed Dr. Lamert, and his celebrated pupil Dr. Brodum, who will be introduced into the valuable pages of your Journal, in the subsequent letter.

In reflecting upon the various transitions experienced by Dr. Mayersbach in his professional character, it is remarkable enough, that several popular characters, after having suddenly lost their reputation, or, from the caprice of fashion, lost their professional employment, have retired a few months; returned after a period of absence to the metropolis, and regained their former practice. Sir Richard Jebb told me, and he had the information from Sir Edward Wilmot, Bart. himself, that two ladies of fashion, near the Court, died under his care (Sir Edward's) at the same period, which was so buzzed about amongst the circles of fashion, as to turn the tide so adversely against him, which had long and deservedly flowed in favour of Sir Edward, that from three thousand pounds a year, his professional income sunk to three hundred. This induced him to take a tour on the continent, from which he returned the next year, and as suddenly regained his former professional employment. This respectable physician retired into Dorsetshire in an advanced age, and died a few years ago, æt. 93. A little before this time, I had a letter from him, written in a good hand, and with great perspicuity; and which is now in the possession of

*London, July* 23, 1804. IETROS.

